# Extracellular Matrix-Based and Extracellular Matrix-Bioinspired Scaffolds for Extracellular Vesicle Delivery in Dental Pulp Regeneration: A Narrative Review

**DOI:** 10.3390/biotech15030054

**Published:** 2026-07-14

**Authors:** Nevena Cvetic, Anđelka Zivaljevic, Kristina Krstic, Suzana Zivanovic, Milos Papic, Natalija Arsenijevic, Miona Glisic, Tamara Milunovic, Renata Petrovic, Milica Popovic

**Affiliations:** 1 Department of Dentistry, Faculty of Medical Sciences, University of Kragujevac, 34000 Kragujevac, Serbia; nev.cvetic@gmail.com (N.C.); andjelkasimic5@gmail.com (A.Z.); krstic.kristinakrstic.kristina@gmail.com (K.K.); suzana.zivanovic@fmn.kg.ac.rs (S.Z.); arsenijevicnatalija@gmail.com (N.A.); mionagrujovic@yahoo.com (M.G.); tamara.vucicevic@yahoo.com (T.M.); milicapopovic75@gmail.com (M.P.); 2 Department of Restorative Dentistry and Endodontics, School of Dental Medicine, University of Belgrade, 11000 Belgrade, Serbia; renatapetrovic4@yahoo.com

**Keywords:** extracellular vesicles, dental pulp regeneration, extracellular matrix, hydrogels

## Abstract

Vital pulp therapy aims to preserve pulp vitality by stimulating reparative processes. However, conventional approaches often result in incomplete tissue regeneration. Extracellular vesicles (EVs) have emerged as promising cell-free therapeutic agents because of their ability to regulate angiogenesis, odontogenesis, and immune responses through the transfer of bioactive molecules. Despite their significant regenerative potential, the clinical application of EVs remains limited by rapid clearance, insufficient local retention, and uncontrolled release following administration. To address these challenges, various extracellular matrix (ECM)-based and ECM-bioinspired scaffolds have been developed as delivery platforms. These scaffolds can provide structural support and enable controlled, localized release of EVs. This narrative review critically evaluates the current evidence regarding scaffold systems as EV delivery platforms for dental pulp regeneration, comparing their biological performance, methodological quality, and translational potential. Across the available studies, scaffold-assisted EV delivery consistently enhanced angiogenesis, odontogenic differentiation, mineralization, and immunomodulation; however, the evidence remains preclinical and is characterized by substantial heterogeneity regarding EV source, isolation and characterization methods, scaffold composition, experimental models, and outcome assessment. Current findings support the feasibility of scaffold-assisted EV delivery for regenerative endodontics, but important challenges remain, including standardization of EV production and characterization, scalable manufacturing, regulatory approval, and demonstration of long-term safety and functional pulp–dentin complex regeneration. Further well-designed translational and clinical studies will be essential before routine clinical implementation can be considered.

## 1. Introduction

Vital pulp therapy is a biologically based approach aimed at preserving pulp vitality by promoting healing and tertiary dentin formation through the application of bioactive materials that stimulate odontoblast-like cell differentiation and matrix deposition [1]. The most common causes of pulp injury include microbial invasion due to dental caries and microleakage, as well as material-induced inflammation. In response, the pulp activates intrinsic reparative mechanisms to restore tissue integrity [2]. However, these processes are typically limited to the formation of irregular tertiary dentin, rather than complete restoration of the native pulp–dentin complex, underscoring the distinction between reparation and regeneration [3].

To overcome these limitations, regenerative endodontics has increasingly adopted tissue engineering strategies based on the interaction of stem cells, bioactive signals, and scaffolds. Due to limitations of stem cell-based therapies, including immunogenicity, ethical concerns, and procedural complexity, cell-free approaches based on extracellular vesicles (EVs), particularly exosomes, have emerged as promising alternatives [4]. EVs comprise a heterogeneous group of membrane-bound nanoparticles released by virtually all cell types and include exosomes, microvesicles, and apoptotic bodies, which differ in their biogenesis, size, molecular composition, and biological functions. In the field of regenerative endodontics, EVs have received particular attention because of their ability to transfer proteins, lipids, mRNA, and miRNAs that regulate tissue repair and immune responses. Because the terminology used to describe EV populations remains inconsistent across the literature, the nomenclature adopted throughout this review follows the original studies whenever possible, while the broader term EVs is used when referring collectively to these heterogeneous vesicle populations in accordance with current international recommendations [5,6].

**Figure 1:**
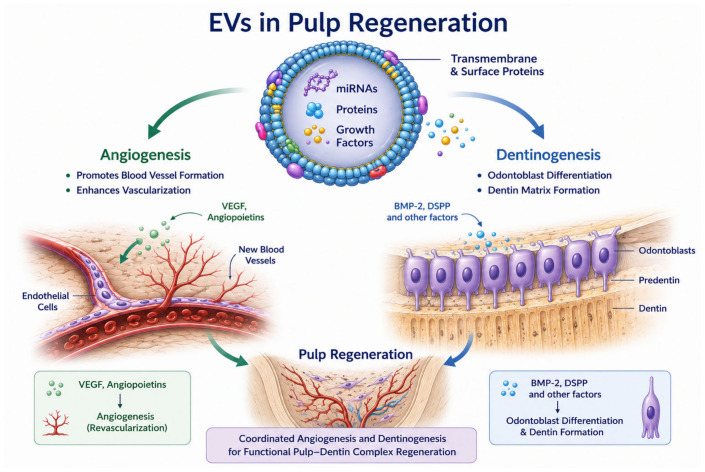
Proposed mechanisms by which EVs contribute to dental pulp regeneration. EVs transport bioactive cargo, including microRNAs (miRNAs), proteins, growth factors, and membrane-associated molecules, which regulate key regenerative processes. Their effects include stimulation of angiogenesis via Vascular Endothelial Growth Factor (VEGF) and angiopoietin-mediated vascularization, as well as promotion of odontogenic differentiation and dentin matrix deposition through Bone Morphogenetic Protein-2 (BMP-2) and DSPP-associated pathways, ultimately supporting regeneration of the pulp–dentin complex. Green arrows indicate angiogenesis-related pathways, whereas blue arrows indicate odontogenic differentiation and dentin formation pathways. This figure was generated using artificial intelligence-assisted tools and subsequently modified and curated by the authors for scientific illustration purposes.

Among the different EV subtypes, exosomes and small EVs (sEVs) have attracted particular attention in regenerative endodontics because of their ability to mediate intercellular communication through the transfer of proteins, lipids, messenger RNAs, microRNAs, and other bioactive molecules [7,8]. Numerous studies have demonstrated that these vesicles regulate key regenerative processes by promoting stem cell proliferation and migration, enhancing angiogenesis, modulating immune responses, and stimulating odontogenic differentiation [9]. In particular, EVs derived from dental pulp stem cells (DPSCs) have been shown to promote revascularization through angiogenic signaling pathways and microRNA-mediated regulation while enhancing odontoblast differentiation by increasing the expression of odontogenic markers such as dentin sialophosphoprotein (DSPP) [10]. Moreover, their immunomodulatory effects, particularly macrophage polarization toward the M2 phenotype, contribute to the establishment of a pro-regenerative microenvironment that supports pulp–dentin complex regeneration [11] (Figure 1). Despite these advantages, their clinical application is limited by rapid degradation, dilution, and insufficient retention at the target site [12].

Biomaterial scaffolds, particularly hydrogel-based systems, have emerged as ideal delivery platforms for EVs due to their structural similarity to the extracellular matrix (ECM), high biocompatibility, and ability to encapsulate and protect bioactive molecules [13,14]. The unique anatomical characteristics of the root canal system make hydrogel-based delivery systems particularly attractive for regenerative endodontics. Injectable hydrogels can completely fill irregular root canal spaces through minimally invasive procedures while providing a hydrated three-dimensional microenvironment that supports nutrient diffusion, cell migration, vascular ingrowth, and localized delivery of therapeutic EVs. Furthermore, their tunable mechanical properties and degradation kinetics allow synchronization of scaffold resorption with newly formed tissue, making them especially suitable for pulp–dentin complex regeneration [3]. Given the growing diversity of biomaterials, scaffold systems may be broadly classified according to their origin and relationship to the native ECM into ECM-based and ECM-bioinspired scaffolds.

Although several recent reviews have discussed EVs in tissue engineering, hydrogel-based delivery systems, regenerative endodontics, or ECM-derived biomaterials, an integrated comparison of ECM-based and ECM-bioinspired scaffolds as EV delivery platforms for dental pulp regeneration, accompanied by a critical appraisal of the current evidence and translational challenges, remains lacking [3,13,14].

The aim of this narrative review is to critically evaluate the current evidence regarding ECM-based and ECM-bioinspired scaffold systems for EV delivery in dental pulp regeneration. Specifically, this review aims to compare the biological performance of different scaffold classes, critically assess the available experimental evidence, discuss the major biological and translational challenges, and highlight future directions for the clinical translation of EV-based regenerative endodontic therapies.

## 2. Literature Search Strategy

This narrative review was conducted through a comprehensive literature search of electronic databases, including PubMed, Scopus, and Web of Science, up to January 2026. The search strategy included combinations of the following keywords: “exosomes”, “extracellular vesicles”, “dental pulp regeneration”, “vital pulp therapy”, “hydrogels”, “scaffolds”, “ECM”, “GelMA”, “alginate”, and “chitosan”.

Only studies published in English were considered. Both in vitro and in vivo preclinical studies, as well as relevant clinical reports, were included. Review articles were screened to identify additional relevant references.

Studies were selected based on their relevance to exosome-based delivery systems and scaffold-assisted regenerative endodontic approaches. Emphasis was placed on studies investigating angiogenesis, odontogenic differentiation, and immunomodulatory effects. Given the increasing recognition of EV heterogeneity, particular attention was paid during data extraction to the cellular origin of EVs, EV terminology, isolation and characterization methods, whenever these data were reported, in accordance with the principles outlined by the International Society for Extracellular Vesicles in the Minimal Information for Studies of Extracellular Vesicles (MISEV) recommendations [6]. Data extraction also included EV cellular source, scaffold composition, biological outcomes, and level of evidence to facilitate critical comparison among studies.

Studies from non-dental tissue engineering, wound healing, or bone regeneration were included only when they provided mechanistic insights relevant to scaffold-assisted EV delivery or biomaterial performance that could inform future applications in dental pulp regeneration. Their indirect nature is explicitly acknowledged throughout the review.

Due to heterogeneity in study design and outcome measures, a qualitative synthesis approach was applied. No formal risk of bias assessment was performed due to the narrative nature of this review.

## 3. ECM-Based and ECM-Bioinspired Scaffolds

Scaffold-assisted delivery has emerged as one of the most promising strategies for overcoming the major limitations associated with the direct administration of EVs, by providing structural support, protecting EV cargo, and enabling localized and sustained release. Biomaterial scaffolds create a favorable microenvironment for tissue regeneration while enhancing the therapeutic efficacy of EV-based therapies [8,13,14].

Given the growing diversity of biomaterial-based delivery platforms investigated for regenerative endodontics, scaffold systems were broadly categorized in this review according to their origin and relationship to the native ECM into two principal groups: ECM-based scaffolds and ECM-bioinspired scaffolds. ECM-based scaffolds are directly derived from natural tissues or biological matrices and retain many of the structural, biochemical, and biological characteristics of the native ECM, including extracellular matrix proteins, glycosaminoglycans, and endogenous bioactive molecules that regulate cell behavior and tissue regeneration [15]. In contrast, ECM-bioinspired scaffolds comprise natural, synthetic, or semi-synthetic biomaterials engineered to reproduce selected structural, mechanical, or biochemical properties of the native ECM while offering greater control over composition, mechanical characteristics, reproducibility, and functional modification [13,14].

This classification provides the conceptual framework for the present review and facilitates systematic comparison of their biological properties, regenerative performance, and translational potential.

### 3.1. ECM-Based Scaffolds

According to the classification adopted in this review, ECM-based scaffolds closely mimic the natural microenvironment and include decellularized extracellular matrix (dECM), treated dentin matrix (TDM), collagen, fibrin, Matrigel, and hyaluronic acid [3,15].

#### 3.1.1. Decellularized Extracellular Matrix (dECM)

dECM preserves the native ECM architecture while removing immunogenic components [15]. It retains structural proteins and growth factors essential for regeneration, supporting DPSC proliferation, migration, and differentiation. The dECM scaffolds investigated for dental pulp regeneration have been derived from different dental tissue sources and fabricated using distinct processing strategies. Yi et al. [16] developed an injectable hydrogel from porcine dental pulp-derived dECM, whereas Tan et al. [17] obtained dECM from decellularized human dental pulp. More recently, Yuan et al. [18] prepared an injectable xenogeneic hydrogel from porcine dental pulp following optimized decellularization and pepsin digestion, preserving collagen-rich ECM architecture together with glycosaminoglycans and tissue-specific bioactive molecules while effectively removing cellular components. These scaffold systems promoted macrophage polarization, odontogenic differentiation, angiogenesis, and regeneration of pulp-like tissue, highlighting the importance of preserving tissue-specific ECM cues. Although studies combining dECM with EVs in dental pulp regeneration remain limited, the preserved ECM architecture provides abundant binding sites for EV retention, suggesting considerable potential for synergistic regenerative effects [15,18].

The biological performance of dECM scaffolds is strongly influenced by both tissue origin and decellularization methodology. Porcine dental pulp provides an abundant and scalable source for dECM production, whereas human dental pulp-derived matrices may more closely reproduce the native pulp microenvironment but are limited by tissue availability. In regenerative endodontics, dECM may be derived from dental pulp, dentin-pulp complexes, or xenogeneic dental tissues, each providing distinct biochemical composition, structural organization, and regenerative potential [19]. Decellularization protocols commonly combine physical, chemical, and enzymatic approaches, including freeze–thaw cycles, detergent-based treatments, and nuclease digestion to effectively remove cellular components while preserving extracellular matrix architecture [20]. Subsequent enzymatic solubilization with pepsin enables fabrication of injectable hydrogels while largely maintaining collagen-rich matrix components and bioactive molecules, thereby improving adaptation to the complex root canal anatomy. However, excessive decellularization may disrupt collagen ultrastructure, reduce glycosaminoglycan and growth factor retention, impair cell adhesion, and diminish the scaffold’s regenerative capacity [21].

Consequently, variability in tissue source, donor characteristics, and decellularization protocols remains a major obstacle to reproducibility and direct comparison among published studies. These factors may substantially influence scaffold composition, mechanical properties, bioactivity, and regenerative outcomes independently of the biomaterial itself [22]. Standardization of tissue sourcing, decellularization procedures, and physicochemical characterization is therefore essential to ensure consistent biological performance and facilitate the future clinical translation of dECM-based regenerative therapies.

#### 3.1.2. Treated Dentin Matrix (TDM)

TDM is a dentin-derived scaffold rich in bioactive proteins and capable of acting as a reservoir for growth factors [23]. TDM-based hydrogels and pastes promote odontogenic differentiation and continuous dentin bridge formation, with degradation synchronized to new tissue deposition [24]. The incorporation of EVs into TDM scaffolds has been associated with improved vascularization, neuralization, and odontogenic differentiation. In a study by Chen Y et al. [25], swine-derived TDM enriched with exosomes derived from dental pulp tissue demonstrated enhanced odontogenic differentiation as well as improved vascularization and neuralization. Wen B et al. [26] investigated the combination of TDM proteins with DPC-derived sEVs for pulp–dentin complex repair. Gene and protein analyses confirmed that the TDM-sEVs complex upregulated odontoblast-related protein expression in DPCs, while in vivo, both TDM with or without sEVs supported the formation of a continuous reparative dentin bridge, with odontoblast-like high columnar cells observed on the pulp side of the new dentin. These findings suggest that TDM represents a promising scaffold for EV-based pulp regeneration [23].

#### 3.1.3. Collagen

As the primary structural protein of dentin ECM, collagen provides excellent biocompatibility and supports cell adhesion and differentiation [27]. Exosomes isolated from MSCs, incorporated with collagen scaffolds, have demonstrated enhanced angiogenesis, odontogenic differentiation, and formation of vascularized pulp-like tissue [28]. A recent study demonstrated that exosome-enriched collagen hydrogels promote pulp–dentin regeneration by upregulating dentin matrix proteins such as DSPP and Dentin Matrix Acidic Phosphoprotein 1 (DMP1) and enhancing reparative dentin formation [28]. However, collagen’s limited mechanical strength remains a drawback, often requiring modification or combination with other materials [22].

#### 3.1.4. Fibrin-Based Scaffolds

Fibrin-based scaffolds, including platelet-rich plasma (PRP) and platelet-rich fibrin (PRF), are widely used due to their biocompatibility, biodegradability, and strong proangiogenic properties [29]. When combined with DPSC-derived EVs, fibrin hydrogels promote rapid neovascularization, sustained EV retention, increased VEGF secretion, and collagen deposition, thereby supporting the early stages of pulp tissue regeneration [30,31]. Although evidence in regenerative endodontics remains limited, platelet-derived biomaterials have attracted increasing interest because of their intrinsic regenerative potential. PRP-derived exosomes have shown proangiogenic and immunomodulatory effects in non-dental regenerative models, whereas the combination of PRF with stem cell-derived exosomes has enhanced tissue regeneration in preclinical bone models, suggesting that similar strategies may warrant investigation in regenerative endodontics [32,33].

#### 3.1.5. Matrigel

Matrigel is a bioactive ECM-derived hydrogel rich in structural proteins and growth factors, providing a highly supportive environment for cell growth and differentiation [34]. When combined with DPSC-derived exosomes, particularly odontogenically induced DPSC-derived exosomes, Matrigel promoted the formation of vascularized and innervated pulp-like tissue with a well-organized odontoblast-like cell layer, demonstrating the ability of the scaffold to preserve and potentiate the biological activity of EV cargo during pulp–dentin complex regeneration [33]. Despite these promising preclinical findings, the clinical translation of Matrigel remains limited because it is derived from mouse sarcoma and exhibits substantial batch-to-batch variability, xenogeneic origin, and chemically undefined composition, all of which complicate standardization and regulatory approval [35].

#### 3.1.6. Hyaluronic Acid (HA)

HA is a naturally occurring ECM component with roles in inflammation regulation, angiogenesis, and tissue repair [27]. HA-based hydrogels are injectable, biocompatible, and adaptable to root canal anatomy, making them suitable for regenerative endodontics. They support cell proliferation, migration, and angiogenesis, while degradation products further enhance pro-angiogenic signaling. Although direct evidence for EV-loaded HA scaffolds in dental pulp regeneration remains lacking, the regenerative potential of HA hydrogels themselves has been demonstrated in clinical practice. A case report described the regenerative endodontic treatment of a necrotic immature maxillary central incisor using HA hydrogel as a scaffold, resulting in continued root development and complete periapical healing after 12 months of follow-up [36,37]. These findings support the clinical feasibility of HA-based scaffold materials; however, they should not be interpreted as evidence for the clinical translation of EV-assisted regenerative therapies, as studies combining HA with EVs are still lacking.

Although ECM-based scaffolds have shown promising biological performance, several limitations remain. Most studies are based on preclinical models, often with significant variability in scaffold composition, exosome source, and evaluation methods. This heterogeneity makes direct comparison difficult and limits reproducibility.

Furthermore, issues such as batch-to-batch variability (particularly in dECM and Matrigel), limited mechanical stability (collagen), and lack of standardized processing protocols represent significant barriers to clinical translation. Consequently, despite their ability to closely recapitulate the native extracellular matrix and support tissue regeneration, ECM-based scaffolds require further methodological standardization, rigorous preclinical validation, and well-designed translational studies before routine clinical application can be considered. A comparative overview of ECM-based scaffold properties, biological effects, and levels of evidence is presented in Table 1.

### 3.2. ECM-Bioinspired Scaffolds

ECM-bioinspired scaffolds replicate key structural and biochemical features of the ECM and include gelatin methacryloyl (GelMA), alginate, self-assembled peptide (SAP) hydrogels, and chitosan-based systems [22,27].

#### 3.2.1. Gelatin Methacryloyl (GelMA)

GelMA is a photo-crosslinkable gelatin derivative with tunable mechanical properties and high biocompatibility [14]. Owing to its porous three-dimensional architecture and controllable degradation profile, GelMA has emerged as a promising hydrogel for regenerative endodontics and EV delivery [14]. In particular, Lu et al. [38] demonstrated that GelMA hydrogels loaded with functional EVs derived from odontogenically induced stem cells from human exfoliated deciduous teeth (SHEDs) promoted the proliferation, and migration of DPSCs in vitro and facilitated dentinogenesis in vivo. Beyond dental applications, GelMA has also been successfully employed as an EV delivery platform in other tissues, including bone regeneration, where sustained EV release promoted osteogenesis through activation of the PI3K/Akt signaling pathway [39].

#### 3.2.2. Alginate

Alginate is a biocompatible polysaccharide capable of forming hydrogels under mild conditions and has attracted considerable interest as a scaffold material for regenerative endodontics because of its biocompatibility and ability to support cell encapsulation [27,40]. Although direct evidence in dental pulp regeneration remains limited, exosome-loaded alginate scaffolds have demonstrated enhanced angiogenesis, collagen organization, and immunomodulatory effects in non-dental tissue regeneration models, including full-thickness skin wound healing [41]. More recently, Fei et al. [42] incorporated hDPSC-derived apoptotic vesicles (ApoVs) into silk fibroin/sodium alginate (SF/SA) hydrogels and demonstrated significantly enhanced angiogenesis, odontogenic differentiation, and pulp regeneration compared with the scaffold alone, suggesting that bioactive vesicle-loaded alginate-based hydrogels represent a promising strategy for regenerative endodontics.

#### 3.2.3. Self-Assembled Peptide (SAP) Hydrogels

SAP forms nanofibrous structures that closely resemble native ECM architecture [43]. A widely studied example is RADA16-I, which forms highly hydrated 3D nanofiber networks closely mimicking native ECM architecture [43]. These materials support odontogenic differentiation, angiogenesis, and formation of pulp-like tissue. Functional modifications further enhance regenerative performance. SAP hydrogels also enable controlled exosome delivery, thereby improving angiogenic and odontogenic outcomes [44,45]. LPS-preconditioned human umbilical cord mesenchymal stem cell-derived sEVs incorporated into a SAP hydrogel loaded onto a collagen sponge promoted odontogenesis and angiogenesis. In addition, the CS/SAPNS scaffold enabled controlled sEVs release while preserving sEVs structural integrity [44].

#### 3.2.4. Chitin and Chitosan

Chitin and chitosan are naturally derived polysaccharides characterized by excellent biocompatibility, biodegradability, and favorable biological properties for tissue engineering applications [46,47]. Chitosan additionally exhibits intrinsic antimicrobial activity and supports cell adhesion and differentiation. Chitosan-based scaffolds enriched with bioactive molecules such as basic fibroblast growth factor have further enhanced angiogenesis and pulp-like tissue formation, although independently of EVs incorporation [48]. Owing to their porous structure and abundant functional groups, chitin- and chitosan-based hydrogels have attracted increasing interest as potential EV delivery platforms. Recent reviews have highlighted their ability to support sustained EV release and preserve EV bioactivity in regenerative applications, although direct evidence in dental pulp regeneration remains limited [49,50]. In regenerative endodontics, Wang et al. [51] demonstrated that an injectable thermosensitive chitin-based hydrogel loaded with hDPSC-derived exosomes significantly enhanced angiogenesis and odontogenic differentiation, supporting the potential of chitin-based EV delivery systems for pulp–dentin complex regeneration.

Compared to native ECM-based scaffolds, ECM-bioinspired materials offer improved tunability, manufacturing reproducibility, and mechanical stability, facilitating scaffold customization for specific regenerative applications. However, they do not fully reproduce the full biological complexity of native ECM, which can influence cellular responses. In addition, the long-term in vivo performance of these materials, particularly with respect to degradation kinetics, host tissue interactions, and immune responses, remains insufficiently investigated. A comparative overview of ECM-bioinspired scaffold properties, biological effects, and levels of evidence is presented in Table 2.

## 4. Discussion

This review highlights the growing importance of EV-loaded scaffold systems as emerging cell-free strategies for dental pulp regeneration. By integrating EVs with biomaterial scaffolds, these delivery platforms may overcome several limitations associated with direct administration of EVs, including rapid clearance, insufficient local retention, and uncontrolled release, while simultaneously providing a biomimetic microenvironment that supports tissue repair and regeneration [37,52]. However, despite the encouraging biological outcomes reported to date, the currently available evidence remains predominantly preclinical and is characterized by considerable methodological heterogeneity regarding EV source, isolation and characterization methods, scaffold composition, experimental models, and outcome assessment. Consequently, the available data should be interpreted cautiously when considering future clinical translation.

A major finding emerging from this review is the complementary role of ECM-based and ECM-bioinspired scaffold systems. Native ECM-derived systems, including dECM, TDM, collagen, fibrin, Matrigel, and hyaluronic acid, most closely reproduce the biochemical complexity of the natural pulp microenvironment. Their intrinsic content of structural proteins and bioactive molecules provides biological cues that support angiogenesis, odontogenic differentiation, immune modulation, and cellular organization, thereby creating favorable conditions for pulp–dentin regeneration. Nevertheless, their regenerative performance is strongly influenced by tissue source, decellularization methodology, and batch-to-batch variability, while limited mechanical stability and difficulties in manufacturing standardization continue to represent major barriers to reproducibility and clinical translation [20,22,53,54].

In contrast, ECM-bioinspired scaffolds, including GelMA, alginate, SAP, and chitin/chitosan-based hydrogel, provide greater control over scaffold architecture, degradation kinetics, mechanical properties, and manufacturing reproducibility. These characteristics facilitate injectable delivery, sustained EV release, and adaptation to the complex anatomy of the root canal system. Although these materials cannot fully reproduce the biological complexity of native ECM, their tunability and manufacturing reproducibility make them attractive candidates for future translational applications [55].

Another important observation emerging from this review is that regenerative outcomes appear to depend not only on the biological activity of EVs but also on the interaction between EVs and their delivery scaffold. Notably, nearly all scaffold systems identified in this review are hydrogel-based, reflecting the unique anatomical and biological requirements of regenerative endodontics. Unlike many other regenerative applications, pulp regeneration occurs within a narrow, enclosed root canal space where successful healing depends on complete adaptation to complex canal geometry, rapid vascular ingrowth, coordinated immune modulation, and homogeneous distribution of regenerative signals. Injectable hydrogels are particularly well suited to these requirements because they simultaneously function as space-filling matrices, temporary extracellular microenvironments, and controlled EV delivery platforms [26,40]. Across the included studies, incorporation of EVs consistently enhanced angiogenesis, odontogenic differentiation, mineralization, and immunomodulation, frequently through stimulation of pro-angiogenic signaling pathways, increased expression of odontogenic markers, and polarization of macrophages toward the anti-inflammatory M2 phenotype [41,56,57]. However, direct comparison among studies remains difficult because scaffold composition, EV source, isolation methods, dosing strategies, and biological endpoints varied substantially. Moreover, although many studies demonstrated enhanced mineralization or expression of regenerative markers, relatively few provided convincing evidence of complete functional regeneration characterized by organized odontoblast-like cell layers, vascularization, innervation, and long-term maintenance of pulp vitality. Therefore, the currently available evidence suggests that scaffold-assisted EV delivery represents a highly promising regenerative strategy, but it remains difficult to determine whether the reported biological outcomes are primarily scaffold-driven, EV-driven, or result from synergistic interactions between both components.

**Figure 2:**
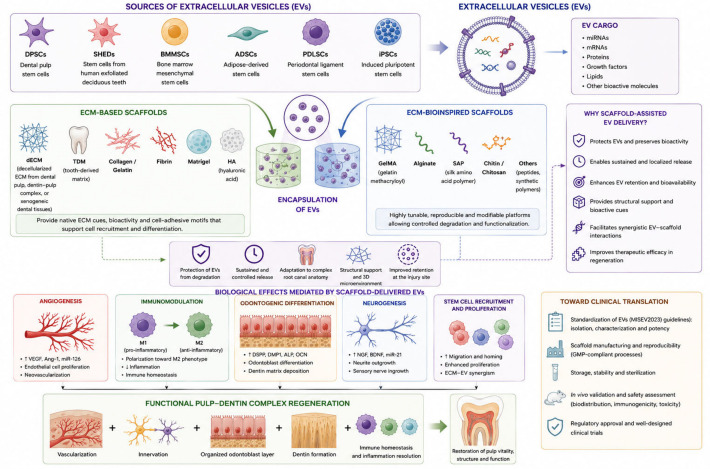
Schematic overview of scaffold-assisted extracellular vesicle (EV) delivery for dental pulp regeneration. ECM-based and ECM-bioinspired scaffolds improve EV retention, protection, and sustained local release within the root canal environment, thereby enhancing the biological activity of EV cargo. Scaffold-mediated EV delivery promotes angiogenesis, immunomodulation, odontogenic differentiation, neurogenesis, and stem cell recruitment, ultimately supporting functional pulp–dentin complex regeneration. The figure also highlights major translational considerations, including standardized EV characterization, scalable manufacturing, and regulatory requirements for future clinical implementation. This figure was generated using artificial intelligence-assisted tools and subsequently modified and curated by the authors for scientific illustration purposes. ↑ indicates an increase or enhancement of the respective biological process or property; ↓ indicates a decrease or reduction of the respective biological process or property.

The principal concepts discussed throughout this section are summarized schematically in Figure 2, illustrating how scaffold properties, EV biology, and engineering strategies collectively contribute to functional pulp–dentin complex regeneration while highlighting the major translational challenges that remain to be addressed.

### 4.1. Comparative Assessment of Scaffold Systems

A critical evaluation of the currently available evidence indicates that not all scaffold systems provide comparable levels of support for EV-mediated dental pulp regeneration. Among ECM-based materials, TDM and dECM appear to possess the strongest biological rationale due to their ability to preserve native odontogenic cues, ECM architecture, and tissue-specific signalling molecules. Studies employing these scaffolds consistently reported enhanced odontogenic differentiation, angiogenesis, and formation of pulp-like tissues, suggesting a greater capacity to recreate the native pulp microenvironment [16,17,18,25,26]. In contrast, collagen and fibrin primarily provide structural support and favorable biological properties for EV retention, whereas Matrigel offers a highly bioactive environment, but its translational potential remains restricted by its tumor origin and batch-to-batch variability [28,30,34]. Hyaluronic acid demonstrates encouraging regenerative properties and clinical feasibility as a scaffold material [36]; however, direct evidence supporting its application as an EV delivery platform in dental pulp regeneration is currently unavailable.

Although these findings suggest differences among ECM-based scaffold systems, direct comparison remains difficult because the available studies vary substantially in scaffold preparation, experimental design, outcome measures, and levels of evidence. Consequently, the relative advantages of individual ECM-based biomaterials cannot yet be established with confidence. Furthermore, the currently available evidence is inherently unbalanced, since only selected ECM-based scaffolds have been directly investigated in combination with EVs, whereas others, such as hyaluronic acid, were included primarily to provide a broader comparative perspective on scaffold performance and translational potential. Moreover, the overall level of evidence remains relatively low. Most studies included in this review combined in vitro experiments with small-animal in vivo models.

Compared with native ECM-based biomaterials, ECM-bioinspired scaffolds provide greater flexibility in scaffold design, allowing precise control over mechanical properties, degradation kinetics, injectability, and EV release profiles [13,14,40]. These characteristics make them particularly attractive for regenerative endodontics, where adaptation to the complex root canal anatomy and sustained local delivery of EVs are essential [13,40]. Nevertheless, the available evidence remains heterogeneous across individual scaffold systems. While GelMA, alginate, SAP hydrogels, and chitin-based scaffolds have all demonstrated encouraging regenerative outcomes following EV incorporation [38,42,43,51], the number of available studies remains limited, and direct comparisons among these biomaterials are currently not possible. Furthermore, not all ECM-bioinspired scaffolds included in this review have been directly evaluated as EV delivery platforms in dental pulp regeneration. For example, chitosan was included because of its favorable biological properties and potential as an EV carrier, although current evidence in dental pulp regeneration is limited to non-EV applications or indirect evidence from other regenerative models [48,49].

The level of experimental evidence also differs considerably among scaffold categories. Most available studies evaluating EV-loaded GelMA, TDM, dECM, fibrin, and chitin-based systems include both in vitro and in vivo investigations, whereas evidence supporting alginate, SAP hydrogels, and especially hyaluronic acid and chitosan is comparatively limited or partially derived from studies without EV incorporation or from non-dental regenerative models. Therefore, extrapolation of these findings to regenerative endodontics should be performed cautiously.

As summarized in Table 1 and Table 2, substantial methodological heterogeneity persists regarding EV source, scaffold composition, isolation protocols, characterization methods, experimental design, and evaluated biological outcomes. Successful regeneration was assessed using diverse endpoints, ranging from angiogenesis and mineralization to odontoblast-like cell differentiation and pulp-like tissue formation, making direct comparisons challenging. Consequently, it remains difficult to determine whether the reported regenerative effects are primarily scaffold-driven, EV-driven, or result from synergistic interactions between both components. Nevertheless, comparison of the currently available evidence allows identification of broader biological trends, translational advantages, and important knowledge gaps that should guide future investigation.

Furthermore, considerable heterogeneity exists in EV isolation and characterization. Although several recent studies followed key MISEV recommendations by combining transmission electron microscopy, nanoparticle tracking analysis, and immunoblotting for EV markers, others relied on more limited characterization strategies, potentially affecting reproducibility and comparability of biological outcomes.

### 4.2. Clinical Translation and Regulatory Challenges

Despite the encouraging regenerative outcomes reported in preclinical studies, several important challenges continue to limit the clinical translation of scaffold-assisted EV delivery for dental pulp regeneration. These limitations extend beyond scaffold performance alone and involve multiple aspects of EV manufacturing, characterization, quality control, regulatory approval, and long-term clinical safety. Consequently, current evidence supports the feasibility of this therapeutic strategy rather than its readiness for routine clinical implementation.

One of the major challenges is the lack of standardized protocols for EV production [58]. Therapeutic efficacy may vary considerably depending on donor-cell source, cell culture conditions, preconditioning strategies, isolation methods, purification procedures, storage conditions, and dosing regimens. These variables directly influence EV yield, molecular cargo, biological activity, and batch-to-batch reproducibility, making comparison among studies difficult and limiting manufacturing consistency [5,6,59]. In addition, purification remains one of the principal bottlenecks for clinical translation. Current isolation techniques differ considerably with respect to EV yield, purity, scalability, and preservation of biological activity, and no universally accepted purification strategy has yet emerged for large-scale clinical production [60].

In regenerative endodontics, EVs may be derived from a variety of cell sources, including DPSCs, SHEDs, bone marrow mesenchymal stem cells, adipose-derived stem cells, and induced pluripotent stem cell-derived populations, among others [50,53]. These cell sources differ in their molecular cargo, regenerative potential, and immunomodulatory properties, which may substantially influence therapeutic efficacy. Although DPSC-derived EVs predominate in the currently available literature because of their tissue-specific regenerative characteristics, direct comparative studies between different donor-cell populations remain scarce [11,53]. Consequently, the optimal EV source for dental pulp regeneration has yet to be established.

Another important consideration is EV characterization. As demonstrated in the present review, substantial methodological heterogeneity exists regarding EV isolation and characterization among the available studies. While many recent investigations combined transmission electron microscopy, nanoparticle tracking analysis, and immunoblotting for EV-associated markers, earlier studies often relied on more limited characterization approaches and frequently used the term exosomes despite incomplete vesicle characterization. Such inconsistencies complicate the interpretation of the literature and emphasize the importance of rigorous EV characterization for future translational research [5,6,56]. Adoption of standardized protocols consistent with current MISEV recommendations will therefore be essential for improving methodological reproducibility, facilitating comparison among studies, and strengthening the quality of future preclinical and clinical research [6,58].

Manufacturing also represents a significant obstacle to clinical implementation [59,60,61]. Production of clinical-grade EVs requires scalable manufacturing processes capable of generating highly reproducible preparations while maintaining biological potency and sterility [59,60,61,62]. In addition, storage conditions, cryopreservation protocols, and long-term stability remain insufficiently standardized and may significantly influence EV integrity and therapeutic efficacy [59,62]. Development of robust manufacturing pipelines compliant with Good Manufacturing Practice (GMP) standards will therefore be necessary before widespread clinical application becomes feasible [59,60,61].

Another unresolved issue concerns EV biodistribution and persistence following administration. Although scaffold incorporation may improve local retention and sustained release, the fate of EVs after release remains incompletely understood. Limited information is available regarding their tissue penetration, cellular uptake, clearance mechanisms, and persistence within regenerated pulp tissues. Advanced imaging and tracking technologies may therefore facilitate future optimization of EV delivery strategies by providing a more comprehensive understanding of EV behavior in vivo [8,58].

Regulatory considerations represent an additional challenge. Unlike conventional biomaterials, scaffold-assisted EV therapies combine biological products with advanced biomaterial delivery systems, resulting in complex regulatory classification and quality-control requirements [59,60,61,62]. Standardized release criteria, potency assays, sterility testing, biodistribution studies, immunogenicity assessment, and long-term safety evaluation will all be required before regulatory approval can be achieved. Furthermore, the absence of clinical trials evaluating EV-loaded scaffolds for dental pulp regeneration currently prevents meaningful assessment of long-term therapeutic efficacy and safety in patients [31,59].

Although EV-based therapies are generally considered safer than cell-based approaches because they eliminate the risks associated with viable cell transplantation, several important safety concerns remain unresolved. These include potential off-target biological effects, unintended immunomodulation, variability in cargo composition, and the possibility of transferring undesirable molecular signals from donor cells. Long-term safety data are particularly limited in regenerative endodontics, emphasizing the need for carefully designed preclinical studies and well-controlled clinical trials before routine clinical implementation can be considered [60,61,63].

Overall, scaffold-assisted EV delivery represents one of the most promising cell-free strategies for regenerative endodontics. However, successful clinical translation will depend not only on further optimization of scaffold design but also on rigorous methodological standardization, reproducible EV manufacturing, comprehensive biological characterization, regulatory harmonization, and well-designed clinical studies capable of demonstrating long-term safety and functional regeneration of the pulp–dentin complex.

### 4.3. Engineering Strategies to Enhance EV-Based Pulp Regeneration

Recent advances in EV engineering offer new opportunities to improve the biological performance of EV-based therapies and overcome several limitations currently restricting their clinical translation. Engineering strategies may be broadly classified into donor-cell engineering, EV cargo engineering, and surface engineering [62]. These strategies may enhance EV cargo composition, biological potency, targeting efficiency, and retention within the regenerative microenvironment, thereby complementing scaffold-assisted delivery approaches. One promising strategy involves the modification of donor cells before EV isolation. Hypoxic preconditioning, inflammatory stimulation, and odontogenic induction have all been shown to influence EV cargo composition and enhance regenerative activity. In regenerative endodontics, donor-cell preconditioning may increase the abundance of pro-angiogenic, odontogenic, neurogenic, and immunomodulatory signaling molecules within EV cargo, thereby improving therapeutic efficacy and potentially enhancing reproducibility [33,62,64].

Additional engineering approaches focus on direct modification of EV cargo through the incorporation of selected microRNAs, proteins, or other bioactive molecules. Such approaches may also reduce biological variability by generating EV preparations with more consistent molecular cargo and predictable biological activity. These strategies may selectively enhance regenerative pathways relevant to pulp repair, including angiogenesis, odontoblast differentiation, neurogenesis, and inflammation resolution. Surface engineering has also been investigated to improve tissue targeting, cellular uptake, and local retention, potentially reducing the EV dose required to achieve therapeutic benefit [62,65]. Although these approaches remain largely experimental, they illustrate how EV engineering could enable more precise regulation of regenerative processes than naturally secreted EV populations.

From a regenerative endodontic perspective, EV engineering may enable customization of biological activity according to the sequential stages of pulp–dentin regeneration. Successful regeneration requires coordinated angiogenesis, immunomodulation, odontogenic differentiation, and ultimately neural integration within the confined root canal environment. Accordingly, EVs enriched with pro-angiogenic cargo may be particularly beneficial during the early stages of tissue repair, whereas EVs carrying odontogenic or neurogenic signaling molecules may support subsequent tissue maturation and functional integration. Such stage-specific approaches could improve the spatial and temporal coordination of regenerative events and ultimately enhance the quality and functionality of regenerated pulp tissue [53,65].

The therapeutic performance of engineered EVs is likely to depend not only on their molecular cargo but also on the properties of the delivery scaffold [13,14,65]. As discussed throughout this review, scaffold composition, porosity, degradation kinetics, mechanical characteristics, and surface chemistry collectively influence EV retention, protection from degradation, release kinetics, and biological activity. Consequently, future scaffold design should move beyond passive EV encapsulation toward multifunctional biomaterials capable of actively regulating EV bioavailability and tissue-specific regenerative signaling. Such integrated scaffold–EV systems may provide more predictable regenerative outcomes than optimization of either component alone.

Despite these promising advances, EV engineering also introduces additional challenges. More extensive manipulation of donor cells or EV cargo may increase manufacturing complexity, production costs, and quality-control requirements while potentially altering EV biodistribution, cellular uptake, and biological safety. Moreover, additional engineering steps may further complicate product standardization and regulatory classification, emphasizing the need for rigorous evaluation before clinical implementation [59,60,61,62].

Overall, EV engineering represents a promising next step in the evolution of cell-free regenerative endodontics by enabling more precise modulation of EV biological activity and improving coordination between scaffold properties and therapeutic cargo. Nevertheless, these approaches remain at an early stage of development, and current evidence is largely limited to experimental studies [53]. Future research should therefore focus on validating engineered EV platforms in clinically relevant orthotopic models, establishing standardized manufacturing and characterization protocols, and determining whether the additional complexity introduced by EV engineering translates into meaningful improvements in long-term functional pulp–dentin complex regeneration.

In conclusion, ECM-based and ECM-bioinspired scaffolds combined with EVs represent one of the most promising cell-free approaches currently being investigated for dental pulp regeneration. Both ECM-based and ECM-bioinspired scaffolds demonstrated encouraging regenerative potential, although they offer distinct advantages. However, no currently available scaffold platform fully satisfies the biological, technological, and regulatory requirements necessary for routine clinical application.

The future of regenerative endodontics will likely depend not on the independent optimization of EVs or biomaterials, but on the rational integration of both components into multifunctional and clinically translatable therapeutic platforms. Achieving this goal will require coordinated advances in EV standardization, scaffold design, manufacturing scalability, regulatory frameworks, and long-term safety assessment. Until these challenges are addressed, EV-loaded scaffold systems should be regarded as highly promising experimental strategies rather than clinically established therapies. Nevertheless, continued progress in bioengineering, regenerative medicine, and translational research may ultimately enable the development of predictable and biologically driven treatments capable of achieving restoration of a functional pulp–dentin complex.

## Figures and Tables

**Table 1:** Overview of original studies investigating ECM-based scaffold for dental pulp regeneration. Studies lacking direct evidence of EV-assisted delivery are included for comparative purposes and are indicated accordingly. For studies incorporating EVs, the reported EV source, isolation method, and characterization approaches are summarized to facilitate critical comparison of methodological heterogeneity in the context of current MISEV2023 recommendations.

Scaffold	Study [Ref]	Key Properties	Application	Main Biological Effect	Exosome Role	Key Outcomes	Level of Evidence	Limitations	EV Source	Isolation Method/Characterization
dECM	Yi P et al. [16]	Injectable, dental pulp-derived hydrogel	Dentin repair	Immunomodulatory	Not applicable	M2 polarization, dentin formation	In vitro	No in vivo validation	Not applicable	Not applicable
	Tan Q et al. [17]	Injectable, dental pulp-derived hydrogel	Pulp regeneration	DPSC proliferation, odontogenesis, angiogenesis	Not applicable	Vascularized pulp-like tissue formation	In vitro/Ectopic In vivo	No orthotopic pulp model; no dentin-like tissue formation;	Not applicable	Not applicable
	Yuan S et al. [18]	Injectable thermoresponsive dental pulp-derived hydrogel	Functional regeneration	Odontogenic	Not applicable	Functional pulp tissue	In vitro/In vivo	Degradation rate	Not applicable	Not applicable
TDM	Chen Y et al. [25]	Tubular dentin-derived ECM scaffold	Pulp regeneration	Odontogenic/angiogenic	Exosome synergy	↑ vascularization, neurogenesis, odontogenesis	In vitro/In vivo	No expression of proteins related to neurogenesis and angiogenesis	DPT and DPC-derived exosomes	DUC, UF, PP/TEM, DLS, WB (CD63, CD9)
	Wen B et al. [26]	Bioactive dentin-derived matrix	Dentin bridge formation	Odontogenic	EV-enhanced signaling	↑ odontoblast markers, reparative dentin	In vitro/In vivo	Variable proliferation	DPC-derived sEVs	UF, PP/TEM, NTA, WB (CD63, HSP70, ALIX)
Collagen	Shi J et al. [28]	Injectable, ECM-like scaffold	Pulp regeneration	Angiogenic/odontogenic	EV carrier	↑ DSPP, DMP1	In vitro/In vivo	Single EV dose evaluated	MSC-derived exosomes	TFF/NTA (ZetaView), CD73 activity, CD81, ALIX, TSG101 (MISEV2018)
Fibrin	Zhang et al. [30]	Injectable, in situ forming	Pulp regeneration (vascularization phase)	Strong angiogenic effect	Sustained EV delivery and retention	Rapid vascular-like structure formation, ↑ VEGF release, ↑ collagen I/III/IV deposition	In vitro	EV yield limitations	DPSC-derived EVs	DUC/TEM, NTA, WB (CD63, TSG101, calnexin)
Matrigel	Wang Y et al. [34]	Injectable hydrogel	Pulp-like tissue formation	Angiogenic/odontogenic	Enhances EV effects	Vascularized pulp-like tissue	In vitro/In vivo	Number of odontoblast-like cells	DPSC-derived exosomes/DPSC-derived Od-exosomes	DUC/TEM, NTA, WB (TSG101, CD63)
HA	Singh H et al. [36]	Injectable hydrogel, bioactive, connective tissue component	Regenerative endodontic therapy	Regenerative	Not applicable	Root development, healing	Clinical	No EV incorporation	Not applicable	Not applicable

Abbreviations: EV—Extracellular vesicle; dECM—Decellularized extracellular matrix; DPSC—Dental pulp stem cells; TDM—Treated dentin matrix; ECM—Extracellular matrix; DPT—Dental pulp tissue; DPC—Dental pulp cells; DUC—Differential ultracentrifugation; UF—Ultrafiltration; PP—Polymer precipitation; TEM—Transmission electron microscopy; DLS—Dynamic light scattering; WB—Western blot; CD – Cluster of differentiation; sEVs—Small extracellular vesicles; NTA—Nanoparticle tracking analysis; HSP—Heat shock protein; ALIX—ALG-2-interacting protein X; TSG—Tumor susceptibility gene; DSPP—Dentin sialophosphoprotein; DMP1—Dentin matrix acidic phosphoprotein 1; MSC—Mesenchymal stem cells; TFF—Tangential flow filtration; MISEV—Minimal information for studies of extracellular vesicles; VEGF—Vascular endothelial growth factor; Od-exosomes—Odontogenic-induced exosomes ↑ indicates an increase or enhancement of the respective biological process or property.

**Table 2:** Overview of original studies investigating ECM-bioinspired scaffold for dental pulp regeneration. Studies lacking direct evidence of EV-assisted delivery are included for comparative purposes and are indicated accordingly. For studies incorporating EVs, the reported EV source, isolation method, and characterization approaches are summarized to facilitate critical comparison of methodological heterogeneity in the context of current MISEV2023 recommendations.

Scaffold	Study [Ref]	Key Properties	Application	Main Biological Effect	Exosome Role	Key Outcomes	Level of Evidence	Limitations	EV Source	Isolation Method/Characterization
GelMA	Lu H et al. [38]	Injectable hydrogel, photo-crosslinkable	Endodontic regeneration	Odontogenic	EV carrier	↑ dentinogenesis	In vitro/ In vivo	Requires polymerization	SHED-derived EVs	DUC/TEM, AFM, NTA, WB (CD63, TSG101)
Alginate SF/SA	Fei Y et al. [42]	Injectable hydrogel, Composite scaffold, freeze-dried scaffold	Regeneration	Angiogenic	EV-loaded system	↑ odontogenesis, angiogenesis and neurogenesis	In vitro/In vivo	No long-term functional evaluation	hDPSC-derived ApoVs	DUC/TEM, NTA, Annexin V, WB (Caspase-3, Cleaved Caspase-3)
Collagen sponge/SAP (CS/SAPNS)	Zeng J et al. [44]	Composite scaffold	Pulp repair	Odontogenic	Controlled release, Maintain the integrity	↑ osteo/odontogenic differentiation	In vitro	No in vivo validation	LPS-hUCMSC-derived sEVs	DUC/TEM, NTA, WB (CD63, CD9, TSG101)
Chitosan (PCL/CS)	Divband B et al. [48]	Porous scaffold, bFGF-loaded	Pulp regeneration	Angiogenic	Not applicable	↑ angiogenesis	In vitro	No in vivo validation	Not applicable	Not applicable
Chitin (HPCH/C)	Wang S et al. [51]	Injectable thermosensitive hydrogel, gelated in situ	Regeneration	Regenerative	EV delivery	↑ odontogenesis, angiogenesis	In vitro/In vivo	Complexity	hDPSC-derived exosomes	DUC/TEM, NTA

Abbreviations: EV—Extracellular vesicle; GelMA—gelatin methacryloyl; SHED—Stem cells from human exfoliated deciduous teeth; DUC—Differential ultracentrifugation; TEM—Transmission electron microscopy; AFM—Atomic force microscopy; NTA—Nanoparticle tracking analysis; WB—Western blot; CD – Cluster of differentiation; TSG—Tumor susceptibility gene; SAP—Self-assembled peptide; SF/SA—Silk fibroin/sodium alginate; hDPSC—Human dental pulp stem cells; ApoVs—Apoptotic vesicles; CS/SAPNS—Collagen sponge/self-assembling peptide nanofiber scaffold; LPS—Lipopolysaccharide; hUCMSC—Human umbilical cord mesenchymal stem cells; sEV—Small extracellular vesicles; PCL/CS—Poly (ε-caprolactone)/chitosan; bFGF—basic Fibroblast growth factor; HPCH/C—Hydroxypropyl chitin/chitin. ↑ indicates an increase or enhancement of the respective biological process or property.

## Data Availability

No new data were created or analyzed in this study. Data sharing is not applicable to this article.

## Abbreviations

The following abbreviations are used in this manuscript:

AFM	Atomic force microscopy
ALIX	ALG-2-interacting protein X
ApoVs	Apoptotic vesicles
bFGF	basic Fibroblast growth factor
BMP-2	Bone morphogenetic protein-2
CD	Cluster of differentiation
CS/SAPNS	Collagen sponge/self-assembling peptide nanofiber scaffold
DMP1	Dentin matrix acidic phosphoprotein 1
dECM	Decellularized extracellular matrix
DLS	Dynamic light scattering
DPC	Dental pulp cell
DPSC	Dental pulp stem cell
DSPP	Dentin sialophosphoprotein
DPT	Dental pulp tissue
DUC	Differential ultracentrifugation
ECM	Extracellular matrix
EV	Extracellular vesicle
GelMA	Gelatin methacryloyl
GMP	Good Manufacturing Practice
HA	Hyaluronic acid
HPCH/C	Hydroxypropyl chitin/chitin
HSP	Heat shock protein
hUCMSC	Human umbilical cord mesenchymal stem cell
LPS	Lipopolysaccharide
miRNA	microRNA
MISEV	Minimal information for studies of extracellular vesicles
MSC	Mesenchymal stem cell
NTA	Nanoparticle tracking analysis
Od-exosomes	Odontogenic-induced exosomes
PCL/CS	Poly(ε-caprolactone)/chitosan
PP	Polymer precipitation
PRP	Platelet-rich plasma
PRF	Platelet-rich fibrin
SAP	Self-assembled peptide
sEVs	Small extracellular vesicles
SF/SA	Silk fibroin/sodium alginate
SHED	Stem cells from human exfoliated deciduous teeth
TDM	Treated dentin matrix
TEM	Transmission electron microscopy
TFF	Tangential flow filtration
TSG	Tumor susceptibility gene
UF	Ultrafiltration
VEGF	Vascular endothelial growth factor
WB	Western blot

## References

[1] Asgary S., Nosrat A. (2025). Vital pulp therapy: Evidence-based techniques and outcomes. Iran. Endod. J..

[2] Ilić J., Radović K., Brković B., Vasić J., Roganović J. (2020). The diabetic dental pulp repair: Involvement of vascular endothelial growth factor and bone morphogenetic protein 2. Srp. Arh. Celok. Lek..

[3] Quigley R.M., Kearney M., Kennedy O.D., Duncan H.F. (2024). Tissue engineering approaches for dental pulp regeneration: The development of novel bioactive materials using pharmacological epigenetic inhibitors. Bioact. Mater..

[4] Miron R.J., Zhang Y. (2024). Understanding exosomes: Part 1—Characterization, quantification and isolation techniques. Periodontology 2000.

[5] Théry C., Witwer K.W., Aikawa E., Alcaraz M.J., Anderson J.D., Andriantsitohaina R., Antoniou A., Arab T., Archer F., Atkin-Smith G.K. (2018). Minimal information for studies of extracellular vesicles 2018 (MISEV2018): A position statement of the International Society for Extracellular Vesicles and update of the MISEV2014 guidelines. J. Extracell. Vesicles.

[6] Welsh J.A., Goberdhan D.C.I., O’Driscoll L., Buzas E.I., Blenkiron C., Bussolati B., Cai H., Di Vizio D., Driedonks T.A.P., Erdbrügger U. (2024). Minimal information for studies of extracellular vesicles (MISEV2023): From basic to advanced approaches. J. Extracell. Vesicles.

[7] Zou Y., Zhou Y., Li G., Dong Y., Hu S. (2025). Clinical applications of extracellular vesicles: Recent advances and emerging trends. Front. Bioeng. Biotechnol..

[8] Ayanji T., Yan N., Jia L., Cheng K. (2026). Non-invasive administration of exosomes. J. Control Release.

[9] Ucuzian A.A., Gassman A.A., East A.T., Greisler H.P. (2010). Molecular mediators of angiogenesis. J. Burn Care Res..

[10] Tian J., Lou Y., Li M., Duan Y., Liu H., Chen C., Qiu Y., Chen W., Pang C., Xiong Y. (2025). Dental follicle stem cell-derived small extracellular vesicles ameliorate pulpitis by reprogramming macrophage metabolism. Bioact. Mater..

[11] Shi Q., Huo N., Wang X., Yang S., Wang J., Zhang T. (2020). Exosomes from Oral Tissue Stem Cells: Biological Effects and Applications. Cell Biosci..

[12] Zhao H., Shang Q., Pan Z., Bai Y., Li Z., Zhang H., Zhang Q., Guo C., Zhang L., Wang Q. (2018). Exosomes from Adipose-Derived Stem Cells Attenuate Adipose Inflammation and Obesity through Polarizing M2 Macrophages and Beiging in White Adipose Tissue. Diabetes.

[13] Villani C., Murugan P., George A. (2024). Exosome-Laden Hydrogels as Promising Carriers for Oral and Bone Tissue Engineering: Insight into Cell-Free Drug Delivery. Int. J. Mol. Sci..

[14] Ju Y., Hu Y., Yang P., Xie X., Fang B. (2022). Extracellular Vesicle-Loaded Hydrogels for Tissue Repair and Regeneration. Mater. Today Bio.

[15] Jin C., Zhang X., Jin Y., Chien P.N., Heo C.Y. (2025). Acellular Extracellular Matrix Scaffolds in Regenerative Medicine: Advances in Decellularization and Clinical Applications. J. Funct. Biomater..

[16] Yi P., Chen S., Zhao Y., Ku W., Lu H., Yu D., Zhao W. (2024). An injectable dental pulp-derived decellularized matrix hydrogel promotes dentin repair through modulation of macrophage response. Biomater. Adv..

[17] Liang Z., Feng Y., Zhang S., Li J., Rao Z., Zhang K., Lai H., Xie Z., Wu F., Wei L. (2026). Decellularized Dental Pulp Matrix Hydrogel Promotes Functional Endodontic Regeneration In Situ. Int. Endod. J..

[18] Yuan S., Yang X., Wang X., Chen J., Tian W., Yang B. (2023). Injectable Xenogeneic Dental Pulp Decellularized Extracellular Matrix Hydrogel Promotes Functional Pulp Regeneration. Int. J. Mol. Sci..

[19] Adanir N., Khurshid Z., Ratnayake J. (2022). The Regenerative Potential of Decellularized Dental Pulp Extracellular Matrix: A Systematic Review. Materials.

[20] Dehghani S., Aghaee Z., Soleymani S., Tafazoli M., Ghabool Y., Tavassoli A. (2024). An overview of the production of tissue extracellular matrix and decellularization process. Cell Tissue Bank..

[21] Moffat D., Ye K., Jin S. (2022). Decellularization for the retention of tissue niches. J. Tissue Eng..

[22] Sequeira D.B., Diogo P., Gomes B.P.F.A., Peça J., Santos J.M.M. (2024). Scaffolds for dentin–pulp complex regeneration. Medicina.

[23] Bi F., Zhang Z., Guo W. (2023). Treated dentin matrix in tissue regeneration: Recent advantages. Pharmaceutics.

[24] Holiel A.A., Mustafa H.M., Sedek E.M. (2023). Biodegradation of injectable treated dentin matrix hydrogel as a novel pulp capping agent for dentin regeneration. BMC Oral Health.

[25] Chen Y., Ma Y., Yang X., Chen J., Yang B., Tian W. (2022). Application of pulp tissue-derived exosomes in pulp regeneration: A novel cell-homing approach. Int. J. Nanomed..

[26] Wen B., Huang Y., Qiu T., Huo F., Xie L., Liao L., Tian W., Guo W. (2021). Reparative dentin formation by dentin matrix proteins and small extracellular vesicles. J. Endod..

[27] Abbass M.M.S., El-Rashidy A.A., Sadek K.M., Moshy S.E., Radwan I.A., Rady D., Dörfer C.E., Fawzy El-Sayed K.M. (2020). Hydrogels and dentin–pulp complex regeneration: From the benchtop to clinical translation. Polymers.

[28] Shi J., Teo K.Y.W., Zhang S., Lai R.C., Rosa V., Tong H.J., Duggal M.S., Lim S.K., Toh W.S. (2023). MSC exosomes enhance dental pulp cell functions and promote pulp–dentin regeneration. Biomater. Biosyst..

[29] Ducret M., Costantini A., Gobert S., Farges J.C., Bekhouche M. (2021). Fibrin-based scaffolds for dental pulp regeneration: From biology to nanotherapeutics. Eur. Cell Mater..

[30] Zhang S., Thiebes A.L., Kreimendahl F., Ruetten S., Buhl E.M., Wolf M., Jockenhoevel S., Apel C. (2020). Extracellular Vesicles-Loaded Fibrin Gel Supports Rapid Neovascularization for Dental Pulp Regeneration. Int. J. Mol. Sci..

[31] Shi X., Hu X., Jiang N., Mao J. (2025). Regenerative endodontic therapy: From laboratory bench to clinical practice. J. Adv. Res..

[32] Saberian M., Davoudi M., Ghafourian A., Moradi-Sardareh H., Taslim Bakhsh F., Afrisham R. (2025). Biogenesis, isolation, and mechanistic applications of platelet-rich plasma and PRP-derived exosomes in diabetic and chronic wound healing: A comprehensive narrative review. Regen. Eng. Transl. Med..

[33] Hilmy F., Dilogo I.H., Reksodiputro M.H., Antarianto R.D., Al Mashur M.I., Adhimulia K.J. (2024). Evaluation of adipose-derived stem cells (ASCs) exosome implantation and platelet-rich fibrin (PRF) on critical long bone defects in Sprague-Dawley rats. Eur. J. Orthop. Surg. Traumatol..

[34] Wang Y., Mao J., Wang Y., Wang R., Jiang N., Hu X., Shi X. (2025). Odontogenic exosomes simulating the developmental microenvironment promote complete regeneration of pulp-dentin complex in vivo. J. Adv. Res..

[35] Kim D., Youn J., Kim J., Lee J., Yoon J., Kim D.S. (2026). From organoid culture to manufacturing: Technologies for reproducible and scalable organoid production. npj Biomed. Innov..

[36] Singh H., Rathee K., Kaur A., Miglani N. (2021). Pulp regeneration in an immature maxillary central incisor using hyaluronic acid hydrogel. Contemp. Clin. Dent..

[37] Xie Y., Guan Q., Guo J., Chen Y., Yin Y., Han X. (2022). Hydrogels for Exosome Delivery in Biomedical Applications. Gels.

[38] Lu H., Mu Q., Ku W., Zheng Y., Yi P., Lin L., Li P., Wang P., Wu J., Yu D. (2024). Functional extracellular vesicles from SHEDs combined with gelatin methacryloyl promote the odontogenic differentiation of DPSCs for pulp regeneration. J. Nanobiotechnol..

[39] Hu W., Xie X., Xu J. (2025). Epimedium-Derived Exosome-Loaded GelMA Hydrogel Enhances MC3T3-E1 Osteogenesis via PI3K/Akt Pathway. Cells.

[40] Islam M.R.R., Islam R., Sano H., Toida Y., Hoshika S., Ahmed H.M.A., Tomokiyo A. (2025). Emerging Trends of Injectable Hydrogels for Vital Pulp Therapy: A Comprehensive Review. Int. Endod. J..

[41] Shafei S., Khanmohammadi M., Heidari R., Ghanbari H., Taghdiri Nooshabadi V., Farzamfar S., Akbariqomi M., Sanikhani N.S., Absalan M., Tavoosidana G. (2020). Exosome loaded alginate hydrogel promotes tissue regeneration in full-thickness skin wounds: An in vivo study. J. Biomed. Mater. Res. A.

[42] Fei Y., Wang X., Ling Z., Jiang Y., Jiang T., Cao L., Wang J. (2025). Angiogenic apoptotic vesicle-laden silk fibroin/sodium alginate hydrogel for pulp regeneration. Mater. Today Bio.

[43] Dzierżyńska M., Sawicka J., Deptuła M., Sosnowski P., Sass P., Peplińska B., Pietralik-Molińska Z., Fularczyk M., Kasprzykowski F., Zieliński J. (2023). Release systems based on self-assembling RADA16-I hydrogels with a signal sequence that improves wound healing processes. Sci. Rep..

[44] Zeng J., Deng H., Li Q., Kang J., Wu Y. (2024). Scaffold-loaded LPS-hUCMSC-sEVs promote osteo/odontogenic differentiation and angiogenic potential of hDPSCs. Tissue Cell.

[45] Han C., Zhang Z., Sun J., Li K., Li Y., Ren C., Meng O., Yang J. (2020). Self-Assembling Peptide-Based Hydrogels in Angiogenesis. Int. J. Nanomed..

[46] Izadi H., Asadi H., Bemani M. (2025). Chitin: Comparison between Main Sources. Front. Mater..

[47] Hisham F., Akmal M.H.M., Ahmad F., Ahmad K., Samat N. (2024). Biopolymer Chitosan: Potential Sources, Extraction Methods, and Emerging Applications. Ain Shams Eng. J..

[48] Divband B., Pouya B., Hassanpour M., Alipour M., Salehi R., Rahbarghazi R., Shahi S., Aghazadeh Z., Aghazadeh M. (2022). Towards Induction of Angiogenesis in Dental Pulp Stem Cells Using Chitosan-Based Hydrogels Releasing Basic Fibroblast Growth Factor. BioMed Res. Int..

[49] Suresh N., Shanmugavadivu A., Selvamurugan N. (2025). Chitosan–Exosome Synergy: Advanced Cell-Free Scaffold Approaches for Bone Tissue Engineering. Int. J. Biol. Macromol..

[50] Umapathy V.R., Natarajan P.M., Swamikannu B. (2025). Regenerative Strategies in Dentistry: Harnessing Stem Cells, Biomaterials and Bioactive Materials for Tissue Repair. Biomolecules.

[51] Wang S., Xing X., Peng W., Huang C., Du Y., Yang H., Zhou J. (2023). Fabrication of an Exosome-Loaded Thermosensitive Chitin-Based Hydrogel for Dental Pulp Regeneration. J. Mater. Chem. B.

[52] Palakurthi S.S., Shah B., Kapre S., Charbe N., Immanuel S., Pasham S., Thalla M., Jain A., Palakurthi S. (2024). A comprehensive review of challenges and advances in exosome-based drug delivery systems. Nanoscale Adv..

[53] Ahmad P., Estrin N., Farshidfar N., Zhang Y., Miron R.J. (2025). Mechanistic Insights into Dental Stem Cell-Derived Exosomes in Regenerative Endodontics. Int. Endod. J..

[54] Elnawam H., Abdallah A., Nouh S., Elbackly R. (2024). Influence of extracellular matrix scaffolds on histological outcomes of regenerative endodontics in experimental animal models: A systematic review. BMC Oral Health.

[55] Su W., Liao C., Liu X. (2025). Angiogenic and Neurogenic Potential of Dental-Derived Stem Cells for Functional Pulp Regeneration: A Narrative Review. Int. Endod. J..

[56] Ahmad P., Estrin N., Farshidfar N., Zhang Y., Miron R.J. (2025). Isolation Methods of Exosomes Derived from Dental Stem Cells. Int. J. Oral Sci..

[57] Talebpour Amiri F., Omraninava M., Shahzamani S., Khodashenas A., Daryakar A., Nasiry D. (2025). Bioactive and degradable collagen-based three-dimensional scaffold encapsulated with adipose mesenchymal stem cell-derived exosomes improved diabetic wound healing. Regen. Ther..

[58] Zhang Y., Lan M., Chen Y. (2024). Minimal Information for Studies of Extracellular Vesicles (MISEV): Ten-Year Evolution (2014–2023). Pharmaceutics.

[59] Zhang J., Pan Y., She P., Rao L. (2026). From bench to bedside: The promise and roadblocks of extracellular vesicle therapeutics. Theranostics.

[60] Ma Y., Dong S., Wu A., Jeong S.D., Lee A.S., Jiang W., Kim B.Y.S. (2026). Engineering Challenges and Translational Opportunities in Emerging Gene Delivery Platforms. Nat. Biomed. Eng..

[61] Ghodasara A., Raza A., Wolfram J., Salomon C., Popat A. (2023). Clinical Translation of Extracellular Vesicles. Adv. Healthc. Mater..

[62] Danilushkina A.A., Emene C.C., Barlev N.A., Gomzikova M.O. (2023). Strategies for Engineering of Extracellular Vesicles. Int. J. Mol. Sci..

[63] Kong L., Zhao G., Wu X., Ma S. (2026). Extracellular Vesicles in Cancer Diagnosis and Therapy: Advances, Challenges, and Prospects for Clinical Translation. Int. J. Mol. Sci..

[64] ArulJothi K.N., Rajendran R.L., Ahn B.-C., Gangadaran P. (2026). Regenerative Therapy at the Crossroads: From Cell-Based to Cell-Free Precision Medicine. Bioengineering.

[65] Ma Y., Dong S., Grippin A.J., Teng L., Lee A.S., Kim B.Y.S., Jiang W. (2025). Engineering therapeutical extracellular vesicles for clinical translation. Trends Biotechnol..

